# Creating visual explanations improves learning

**DOI:** 10.1186/s41235-016-0031-6

**Published:** 2016-12-07

**Authors:** Eliza Bobek, Barbara Tversky

**Affiliations:** 1 University of Massachusetts Lowell, Lowell, MA USA; 2 Stanford University, Columbia University Teachers College, New York, NY USA

**Keywords:** Learning, Visual communication, STEM, Spatial ability, Dynamic system, Diagrammatic reasoning, Structure, Process, Complex system

## Abstract

Many topics in science are notoriously difficult for students to learn. Mechanisms and processes outside student experience present particular challenges. While instruction typically involves visualizations, students usually explain in words. Because visual explanations can show parts and processes of complex systems directly, creating them should have benefits beyond creating verbal explanations. We compared learning from creating visual or verbal explanations for two STEM domains, a mechanical system (bicycle pump) and a chemical system (bonding). Both kinds of explanations were analyzed for content and learning assess by a post-test. For the mechanical system, creating a visual explanation increased understanding particularly for participants of low spatial ability. For the chemical system, creating both visual and verbal explanations improved learning without new teaching. Creating a visual explanation was superior and benefitted participants of both high and low spatial ability. Visual explanations often included crucial yet invisible features. The greater effectiveness of visual explanations appears attributable to the checks they provide for completeness and coherence as well as to their roles as platforms for inference. The benefits should generalize to other domains like the social sciences, history, and archeology where important information can be visualized. Together, the findings provide support for the use of learner-generated visual explanations as a powerful learning tool.

**Electronic supplementary material:**

The online version of this article (doi:10.1186/s41235-016-0031-6) contains supplementary material, which is available to authorized users.

## Significance

Uncovering cognitive principles for effective teaching and learning is a central application of cognitive psychology. Here we show: (1) creating explanations of STEM phenomena improves learning without additional teaching; and (2) creating visual explanations is superior to creating verbal ones. There are several notable differences between visual and verbal explanations; visual explanations map thought more directly than words and provide checks for completeness and coherence as well as a platform for inference, notably from structure to process. Extensions of the technique to other domains should be possible. Creating visual explanations is likely to enhance students’ spatial thinking skills, skills that are increasingly needed in the contemporary and future world.

Dynamic systems such as those in science and engineering, but also in history, politics, and other domains, are notoriously difficult to learn (e.g. Chi, DeLeeuw, Chiu, & Lavancher, 1994; Hmelo-Silver & Pfeffer, 2004; Johnstone, 1991; Perkins & Grotzer, 2005). Mechanisms, processes, and behavior of complex systems present particular challenges. Learners must master not only the individual components of the system or process (structure) but also the interactions and mechanisms (function), which may be complex and frequently invisible. If the phenomena are macroscopic, sub-microscopic, or abstract, there is an additional level of difficulty. Although the teaching of STEM phenomena typically relies on visualizations, such as pictures, graphs, and diagrams, learning is typically revealed in words, both spoken and written. Visualizations have many advantages over verbal explanations for teaching; can creating visual explanations promote learning?

## Background

### Learning from visual representations in STEM

Given the inherent challenges in teaching and learning complex or invisible processes in science, educators have developed ways of representing these processes to enable and enhance student understanding. External visual representations, including diagrams, photographs, illustrations, flow charts, and graphs, are often used in science to both illustrate and explain concepts (e.g., Hegarty, Carpenter, & Just, 1990; Mayer, 1989). Visualizations can directly represent many structural and behavioral properties. They also help to draw inferences (Larkin & Simon, 1987), find routes in maps (Levine, 1982), spot trends in graphs (Kessell & Tversky, 2011; Zacks & Tversky, 1999), imagine traffic flow or seasonal changes in light from architectural sketches (e.g. Tversky & Suwa, 2009), and determine the consequences of movements of gears and pulleys in mechanical systems (e.g. Hegarty & Just, 1993; Hegarty, Kriz, & Cate, 2003). The use of visual elements such as arrows is another benefit to learning with visualizations. Arrows are widely produced and comprehended as representing a range of kinds of forces as well as changes over time (e.g. Heiser & Tversky, 2002; Tversky, Heiser, MacKenzie, Lozano, & Morrison, 2007). Visualizations are thus readily able to depict the parts and configurations of systems; presenting the same content via language may be more difficult. Although words can describe spatial properties, because the correspondences of meaning to language are purely symbolic, comprehension and construction of mental representations from descriptions is far more effortful and error prone (e.g. Glenberg & Langston, 1992; Hegarty & Just, 1993; Larkin & Simon, 1987; Mayer, 1989). Given the differences in how visual and verbal information is processed, how learners draw inferences and construct understanding in these two modes warrants further investigation.

## Benefits of generating explanations

Learner-generated explanations of scientific phenomena may be an important learning strategy to consider beyond the utility of learning from a provided external visualization. Explanations convey information about concepts or processes with the goal of making clear and comprehensible an idea or set of ideas. Explanations may involve a variety of elements, such as the use of examples and analogies (Roscoe & Chi, 2007). When explaining something new, learners may have to think carefully about the relationships between elements in the process and prioritize the multitude of information available to them. Generating explanations may require learners to reorganize their mental models by allowing them to make and refine connections between and among elements and concepts. Explaining may also help learners metacognitively address their own knowledge gaps and misconceptions.

Many studies have shown that learning is enhanced when students are actively engaged in creative, generative activities (e.g. Chi, 2009; Hall, Bailey, & Tillman, 1997). Generative activities have been shown to benefit comprehension of domains involving invisible components, including electric circuits (Johnson & Mayer, 2010) and the chemistry of detergents (Schwamborn, Mayer, Thillmann, Leopold, & Leutner, 2010). Wittrock’s (1990) generative theory stresses the importance of learners actively constructing and developing relationships. Generative activities require learners to select information and choose how to integrate and represent the information in a unified way. When learners make connections between pieces of information, knowledge, and experience, by generating headings, summaries, pictures, and analogies, deeper understanding develops.

The information learners draw upon to construct their explanations is likely important. For example, Ainsworth and Loizou (2003) found that asking participants to self-explain with a diagram resulted in greater learning than self-explaining from text. How might learners explain with physical mechanisms or materials with multi-modal information?

## Generating visual explanations

Learner-generated visualizations have been explored in several domains. Gobert and Clement (1999) investigated the effectiveness of student-generated diagrams versus student-generated summaries on understanding plate tectonics after reading an expository text. Students who generated diagrams scored significantly higher on a post-test measuring spatial and causal/dynamic content, even though the diagrams contained less domain-related information. Hall et al. (1997) showed that learners who generated their own illustrations from text performed equally as well as learners provided with text and illustrations. Both groups outperformed learners only provided with text. In a study concerning the law of conservation of energy, participants who generated drawings scored higher on a post-test than participants who wrote their own narrative of the process (Edens & Potter, 2003). In addition, the quality and number of concept units present in the drawing/science log correlated with performance on the post-test. Van Meter (2001) found that drawing while reading a text about Newton’s Laws was more effective than answering prompts in writing.

One aspect to explore is whether visual and verbal productions contain different types of information. Learning advantages for the generation of visualizations could be attributed to learners’ translating across modalities, from a verbal format into a visual format. Translating verbal information from the text into a visual explanation may promote deeper processing of the material and more complete and comprehensive mental models (Craik & Lockhart, 1972). Ainsworth and Iacovides (2005) addressed this issue by asking two groups of learners to self-explain while learning about the circulatory system of the human body. Learners given diagrams were asked to self-explain in writing and learners given text were asked to explain using a diagram. The results showed no overall differences in learning outcomes, however the learners provided text included significantly more information in their diagrams than the other group. Aleven and Koedinger (2002) argue that explanations are most helpful if they can integrate visual and verbal information. Translating across modalities may serve this purpose, although translating is not necessarily an easy task (Ainsworth, Bibby, & Wood, 2002).

It is important to remember that not all studies have found advantages to generating explanations. Wilkin (1997) found that directions to self-explain using a diagram hindered understanding in examples in physical motion when students were presented with text and instructed to draw a diagram. She argues that the diagrams encouraged learners to connect familiar but unrelated knowledge. In particular, “low benefit learners” in her study inappropriately used spatial adjacency and location to connect parts of diagrams, instead of the particular properties of those parts. Wilkin argues that these learners are novices and that experts may not make the same mistake since they have the skills to analyze features of a diagram according to their relevant properties. She also argues that the benefits of self-explaining are highest when the learning activity is constrained so that learners are limited in their possible interpretations. Other studies that have not found a learning advantage from generating drawings have in common an absence of support for the learner (Alesandrini, 1981; Leutner, Leopold, & Sumfleth, 2009). Another mediating factor may be the learner’s spatial ability.

## The role of spatial ability

Spatial thinking involves objects, their size, location, shape, their relation to one another, and how and where they move through space. How then, might learners with different levels of spatial ability gain structural and functional understanding in science and how might this ability affect the utility of learner-generated visual explanations? Several lines of research have sought to explore the role of spatial ability in learning science. Kozhevnikov, Hegarty, and Mayer (2002) found that low spatial ability participants interpreted graphs as pictures, whereas high spatial ability participants were able to construct more schematic images and manipulate them spatially. Hegarty and Just (1993) found that the ability to mentally animate mechanical systems correlated with spatial ability, but not verbal ability. In their study, low spatial ability participants made more errors in movement verification tasks. Leutner et al. (2009) found no effect of spatial ability on the effectiveness of drawing compared to mentally imagining text content. Mayer and Sims (1994) found that spatial ability played a role in participants’ ability to integrate visual and verbal information presented in an animation. The authors argue that their results can be interpreted within the context of dual-coding theory. They suggest that low spatial ability participants must devote large amounts of cognitive effort into building a visual representation of the system. High spatial ability participants, on the other hand, are more able to allocate sufficient cognitive resources to building referential connections between visual and verbal information.

## Benefits of testing

Although not presented that way, creating an explanation could be regarded as a form of testing. Considerable research has documented positive effects of testing on learning. Presumably taking a test requires retrieving and sometimes integrating the learned material and those processes can augment learning without additional teaching or study (e.g. Roediger & Karpicke, 2006; Roediger, Putnam, & Smith, 2011; Wheeler & Roediger, 1992). Hausmann and Vanlehn (2007) addressed the possibility that generating explanations is beneficial because learners merely spend more time with the content material than learners who are not required to generate an explanation. In their study, they compared the effects of using instructions to self-explain with instructions to merely paraphrase physics (electrodynamics) material. Attending to provided explanations by paraphrasing was not as effective as generating explanations as evidenced by retention scores on an exam 29 days after the experiment and transfer scores within and across domains. Their study concludes, “the important variable for learning was the *process* of producing an explanation” (p. 423). Thus, we expect benefits from creating either kind of explanation but for the reasons outlined previously, we expect larger benefits from creating visual explanations.

## Present experiments

This study set out to answer a number of related questions about the role of learner-generated explanations in learning and understanding of invisible processes. (1) Do students learn more when they generate visual or verbal explanations? We anticipate that learning will be greater with the creation of visual explanations, as they encourage completeness and the integration of structure and function. (2) Does the inclusion of structural and functional information correlate with learning as measured by a post-test? We predict that including greater counts of information, particularly invisible and functional information, will positively correlate with higher post-test scores. (3) Does spatial ability predict the inclusion of structural and functional information in explanations, and does spatial ability predict post-test scores? We predict that high spatial ability participants will include more information in their explanations, and will score higher on post-tests.

## Experiment 1

The first experiment examines the effects of creating visual or verbal explanations on the comprehension of a bicycle tire pump’s operation in participants with low and high spatial ability. Although the pump itself is not invisible, the components crucial to its function, notably the inlet and outlet valves, and the movement of air, are located inside the pump. It was predicted that visual explanations would include more information than verbal explanations, particularly structural information, since their construction encourages completeness and the production of a whole mechanical system. It was also predicted that functional information would be biased towards a verbal format, since much of the function of the pump is hidden and difficult to express in pictures. Finally, it was predicted that high spatial ability participants would be able to produce more complete explanations and would thus also demonstrate better performance on the post-test. Explanations were coded for structural and functional content, essential features, invisible features, arrows, and multiple steps.

## Method

### Participants

Participants were 127 (59 female) seventh and eighth grade students, aged 12–14 years, enrolled in an independent school in New York City. The school’s student body is 70% white, 30% other ethnicities. Approximately 25% of the student body receives financial aid. The sample consisted of three class sections of seventh grade students and three class sections of eighth grade students. Both seventh and eighth grade classes were integrated science (earth, life, and physical sciences) and students were not grouped according to ability in any section. Written parental consent was obtained by means of signed informed consent forms. Each participant was randomly assigned to one of two conditions within each class. There were 64 participants in the visual condition explained the bicycle pump’s function by drawing and 63 participants explained the pump’s function by writing.

### Materials

The materials consisted of a 12-inch Spalding bicycle pump, a blank 8.5 × 11 in. sheet of paper, and a post-test (Additional file 1). The pump’s chamber and hose were made of clear plastic; the handle and piston were black plastic. The parts of the pump (e.g. inlet valve, piston) were labeled.

Spatial ability was assessed using the Vandenberg and Kuse (1978) mental rotation test (MRT). The MRT is a 20-item test in which two-dimensional drawings of three-dimensional objects are compared. Each item consists of one “target” drawing and four drawings that are to be compared to the target. Two of the four drawings are rotated versions of the target drawing and the other two are not. The task is to identify the two rotated versions of the target. A score was determined by assigning one point to each question if both of the correct rotated versions were chosen. The maximum score was 20 points.

The post-test consisted of 16 true/false questions printed on a single sheet of paper measuring 8.5 × 11 in. Half of the questions related to the structure of the pump and the other half related to its function. The questions were adapted from Heiser and Tversky (2002) in order to be clear and comprehensible for this age group.

### Procedure

The experiment was conducted over the course of two non-consecutive days during the normal school day and during regularly scheduled class time. On the first day, participants completed the MRT as a whole-class activity. After completing an untimed practice test, they were given 3 min for each of the two parts of the MRT. On the second day, occurring between two and four days after completing the MRT, participants were individually asked to study an actual bicycle tire pump and were then asked to generate explanations of its function. The participants were tested individually in a quiet room away from the rest of the class. In addition to the pump, each participant was one instruction sheet and one blank sheet of paper for their explanations. The post-test was given upon completion of the explanation. The instruction sheet was read aloud to participants and they were instructed to read along. The first set of instructions was as follows: “A bicycle pump is a mechanical device that pumps air into bicycle tires. First, take this bicycle pump and try to understand how it works. Spend as much time as you need to understand the pump.” The next set of instructions differed for participants in each condition. The instructions for the visual condition were as follows: “Then, we would like you to draw your own diagram or set of diagrams that explain how the bike pump works. Draw your explanation so that someone else who has not seen the pump could understand the bike pump from your explanation. Don’t worry about the artistic quality of the diagrams; in fact, if something is hard for you to draw, you can explain what you would draw. What’s important is that the explanation should be primarily visual, in a diagram or diagrams.” The instructions for the verbal condition were as follows: “Then, we would like you to write an explanation of how the bike pump works. Write your explanation so that someone else who has not seen the pump could understand the bike pump from your explanation.” All participants then received these instructions: “You may not use the pump while you create your explanations. Please return it to me when you are ready to begin your explanation. When you are finished with the explanation, you will hand in your explanation to me and I will then give you 16 true/false questions about the bike pump. You will not be able to look at your explanation while you complete the questions.” Study and test were untimed. All students finished within the 45-min class period.

## Results

### Spatial ability

The mean score on the MRT was 10.56, with a median of 11. Boys scored significantly higher (M = 13.5, SD = 4.4) than girls (M = 8.8, SD = 4.5), F(1, 126) = 19.07, *p* < 0.01, a typical finding (Voyer, Voyer, & Bryden, 1995). Participants were split into high or low spatial ability by the median. Low and high spatial ability participants were equally distributed in the visual and verbal groups.

### Learning outcomes

**Fig. 1:**
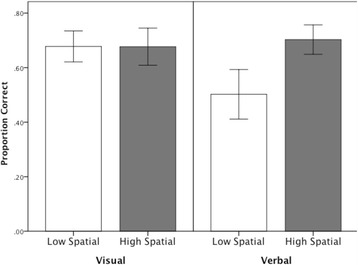
Scores on the post-test by condition and spatial ability

It was predicted that high spatial ability participants would be better able to mentally animate the bicycle pump system and therefore score higher on the post-test and that post-test scores would be higher for those who created visual explanations. Table 1 shows the scores on the post-test by condition and spatial ability. A two-way factorial ANOVA revealed marginally significant main effect of spatial ability F(1, 124) = 3.680, *p* = 0.06, with high spatial ability participants scoring higher on the post-test. There was also a significant interaction between spatial ability and explanation type F(1, 124) = 4.094, *p* < 0.01, see Fig. 1. Creating a visual explanation of the bicycle pump selectively helped low spatial participants.

**Table 1:** Post-test scores, by explanation type and spatial ability

	Explanation type					
	Visual		Verbal		Total	
Spatial ability	Mean	SD	Mean	SD	Mean	SD
Low	11.45	1.93	9.75	2.31	10.60	2.27
High	11.20	1.47	11.60	1.80	11.42	1.65
Total	11.3	1.71	10.74	2.23

### Coding explanations

**Fig. 2:**
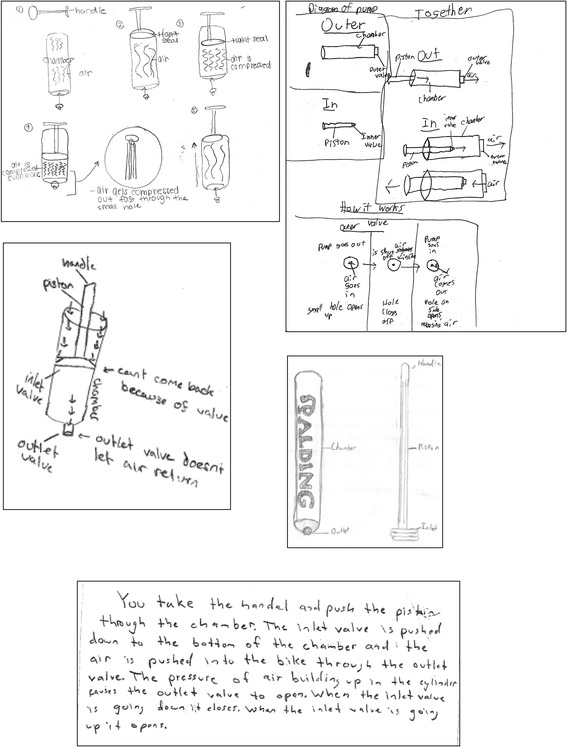
Examples of visual and verbal explanations of the bicycle pump

Explanations (see Fig. 2) were coded for structural and functional content, essential features, invisible features, arrows, and multiple steps. A subset of the explanations (20%) was coded by the first author and another researcher using the same coding system as a guide. The agreement between scores was above 90% for all measures. Disagreements were resolved through discussion. The first author then scored the remaining explanations.

#### Coding for structure and function

A maximum score of 12 points was awarded for the inclusion and labeling of six structural components: chamber, piston, inlet valve, outlet valve, handle, and hose. For the visual explanations, 1 point was given for a component drawn correctly and 1 additional point if the component was labeled correctly. For verbal explanations, sentences were divided into propositions, the smallest unit of meaning in a sentence. Descriptions of structural location e.g. “at the end of the piston is the inlet valve,” or of features of the components, e.g. the shape of a part, counted as structural components. Information was coded as functional if it depicted (typically with an arrow) or described the function/movement of an individual part, or the way multiple parts interact. No explanation contained more than ten functional units.

**Fig. 3:**
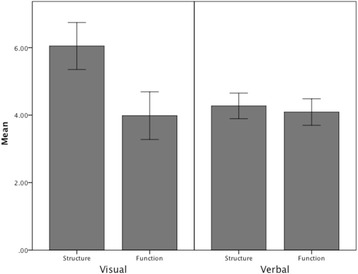
Average number of structural and functional components in visual and verbal explanations

**Fig. 4:**
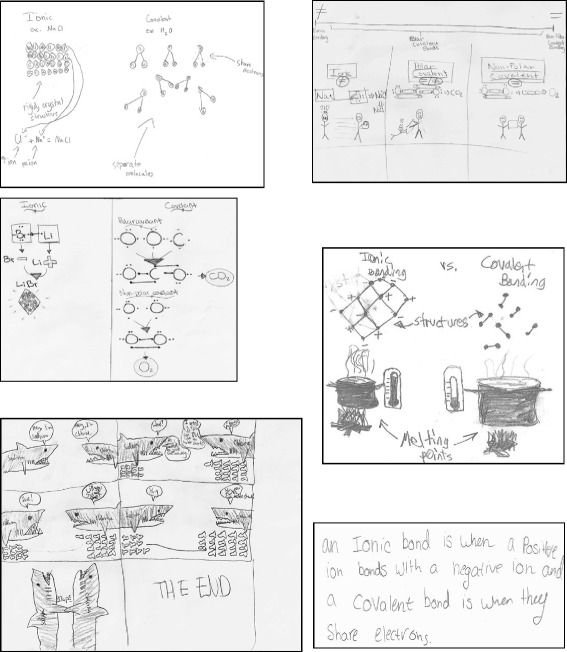
Visual and verbal explanations of chemical bonding

Visual explanations contained significantly more structural components (M = 6.05, SD = 2.76) than verbal explanations (M = 4.27, SD = 1.54), F(1, 126) = 20.53, *p* < 0.05. The number of functional components did not differ between visual and verbal explanations as displayed in Figs. 3 and 4. Many visual explanations (67%) contained verbal components; the structural and functional information in explanations was coded as depictive or descriptive. Structural and functional information were equally likely to be expressed in words or pictures in visual explanations. It was predicted that explanations created by high spatial participants would include more functional information. However, there were no significant differences found between low spatial (M = 5.15, SD = 2.21) and high spatial (M = 4.62, SD = 2.16) participants in the number of structural units or between low spatial (M = 3.83, SD = 2.51) and high spatial (M = 4.10, SD = 2.13) participants in the number of functional units.

#### Coding of essential features

To further establish a relationship between the explanations generated and outcomes on the post-test, explanations were also coded for the inclusion of information essential to its function according to a 4-point scale (adapted from Hall et al., 1997). One point was given if both the inlet and the outlet valve were clearly present in the drawing or described in writing, 1 point was given if the piston inserted into the chamber was shown or described to be airtight, and 1 point was given for each of the two valves if they were shown or described to be opening/closing in the correct direction.

Visual explanations contained significantly more essential information (M = 1.78, SD = 1.0) than verbal explanations (M = 1.20, SD = 1.21), F(1, 126) = 7.63, *p* < 0.05. Inclusion of essential features correlated positively with post-test scores, r = 0.197, *p* < 0.05).

#### Coding arrows and multiple steps

For the visual explanations, three uses of arrows were coded and tallied: labeling a part or action, showing motion, or indicating sequence. Analysis of visual explanations revealed that 87% contained arrows. No significant differences were found between low and high spatial participants’ use of arrows to label and no signification correlations were found between the use of arrows and learning outcomes measured on the post-test.

The explanations were coded for the number of discrete steps used to explain the process of using the bike pump. The number of steps used by participants ranged from one to six. Participants whose explanations, whether verbal or visual, contained multiple steps scored significantly higher (M = 0.76, SD = 0.18) on the post-test than participants whose explanations consisted of a single step (M = 0.67, SD = 0.19), F(1, 126) = 5.02, *p* < 0.05.

#### Coding invisible features

The bicycle tire pump, like many mechanical devices, contains several structural features that are hidden or invisible and must be inferred from the function of the pump. For the bicycle pump the invisible features are the inlet and outlet valves and the three phases of movement of air, entering the pump, moving through the pump, exiting the pump. Each feature received 1 point for a total of 5 possible points.

The mean score for the inclusion of invisible features was 3.26, SD = 1.25. The data were analyzed using linear regression and revealed that the total score for invisible parts significantly predicted scores on the post-test, F(1, 118) = 3.80, *p* = 0.05.

## Discussion

In the first experiment, students learned the workings of a bicycle pump from interacting with an actual pump and creating a visual or verbal explanation of its function. Understanding the functionality of a bike pump depends on the actions and consequences of parts that are not visible. Overall, the results provide support for the use of learner-generated visual explanations in developing understanding of a new scientific system. The results show that low spatial ability participants were able to learn as successfully as high spatial ability participants when they first generated an explanation in a visual format.

Visual explanations may have led to greater understanding for a number of reasons. As discussed previously, visual explanations encourage completeness. They force learners to decide on the size, shape, and location of parts/objects. Understanding the “hidden” function of the invisible parts is key to understanding the function of the entire system and requires an understanding of how both the visible and invisible parts interact. The visual format may have been able to elicit components and concepts that are invisible and difficult to integrate into the formation of a mental model. The results show that including more of the essential features and showing multiple steps correlated with superior test performance. Understanding the bicycle pump requires understanding how all of these components are connected through movement, force, and function. Many (67%) of the visual explanations also contained written components to accompany their explanation. Arguably, some types of information may be difficult to depict visually and verbal language has many possibilities that allow for specificity. The inclusion of text as a complement to visual explanations may be key to the success of learner-generated explanations and the development of understanding.

A limitation of this experiment is that participants were not provided with detailed instructions for completing their explanations. In addition, this experiment does not fully clarify the role of spatial ability, since high spatial participants in the visual and verbal groups demonstrated equivalent knowledge of the pump on the post-test. One possibility is that the interaction with the bicycle pump prior to generating explanations was a sufficient learning experience for the high spatial participants. Other researchers (e.g. Flick, 1993) have shown that hands-on interactive experiences can be effective learning situations. High spatial ability participants may be better able to imagine the movement and function of a system (e.g. Hegarty, 1992).

Experiment 1 examined learning a mechanical system with invisible (hidden) parts. Participants were introduced to the system by being able to interact with an actual bicycle pump. While we did not assess participants’ prior knowledge of the pump with a pre-test, participants were randomly assigned to each condition. The findings have promising implications for teaching. Creating visual explanations should be an effective way to improve performance, especially in low spatial students. Instructors can guide the creation of visual explanations toward the features that augment learning. For example, students can be encouraged to show every step and action and to focus on the essential parts, even if invisible. The coding system shows that visual explanations can be objectively evaluated to provide feedback on students’ understanding. The utility of visual explanations may differ for scientific phenomena that are more abstract, or contain elements that are invisible due to their scale. Experiment 2 addresses this possibility by examining a sub-microscopic area of science: chemical bonding.

## Experiment 2

In this experiment, we examine visual and verbal explanations in an area of chemistry: ionic and covalent bonding. Chemistry is often regarded as a difficult subject; one of the essential or inherent features of chemistry which presents difficulty is the interplay between the macroscopic, sub-microscopic, and representational levels (e.g. Bradley & Brand, 1985; Johnstone, 1991; Taber, 1997). In chemical bonding, invisible components engage in complex processes whose scale makes them impossible to observe. Chemists routinely use visual representations to investigate relationships and move between the observable, physical level and the invisible particulate level (Kozma, Chin, Russell, & Marx, 2002). Generating explanations in a visual format may be a particularly useful learning tool for this domain.

For this topic, we expect that creating a visual rather than verbal explanation will aid students of both high and low spatial abilities. Visual explanations demand completeness; they were predicted to include more information than verbal explanations, particularly structural information. The inclusion of functional information should lead to better performance on the post-test since understanding how and why atoms bond is crucial to understanding the process. Participants with high spatial ability may be better able to explain function since the sub-microscopic nature of bonding requires mentally imagining invisible particles and how they interact. This experiment also asks whether creating an explanation per se can increase learning in the absence of additional teaching by administering two post-tests of knowledge, one immediately following instruction but before creating an explanation and one after creating an explanation. The scores on this immediate post-test were used to confirm that the visual and verbal groups were equivalent prior to the generation of explanations. Explanations were coded for structural and functional information, arrows, specific examples, and multiple representations. Do the acts of selecting, integrating, and explaining knowledge serve learning even in the absence of further study or teaching?

## Method

### Participants

Participants were 126 (58 female) eighth grade students, aged 13–14 years, with written parental consent and enrolled in the same independent school described in Experiment 1. None of the students previously participated in Experiment 1. As in Experiment 1, randomization occurred within-class, with participants assigned to either the visual or verbal explanation condition.

### Materials

The materials consisted of the MRT (same as Experiment 1), a video lesson on chemical bonding, two versions of the instructions, the immediate post-test, the delayed post-test, and a blank page for the explanations. All paper materials were typed on 8.5 × 11 in. sheets of paper. Both immediate and delayed post-tests consisted of seven multiple-choice items and three free-response items. The video lesson on chemical bonding consisted of a video that was 13 min 22 s. The video began with a brief review of atoms and their structure and introduced the idea that atoms combine to form molecules. Next, the lesson showed that location in the periodic table reveals the behavior and reactivity of atoms, in particular the gain, loss, or sharing of electrons. Examples of atoms, their valence shell structure, stability, charges, transfer and sharing of electrons, and the formation of ionic, covalent, and polar covalent bonds were discussed. The example of NaCl (table salt) was used to illustrate ionic bonding and the examples of O_2_ and H_2_O (water) were used to illustrate covalent bonding. Information was presented verbally, accompanied by drawings, written notes of keywords and terms, and a color-coded periodic table.

### Procedure

On the first of three non-consecutive school days, participants completed the MRT as a whole-class activity. On the second day (occurring between two and three days after completing the MRT), participants viewed the recorded lesson on chemical bonding. They were instructed to pay close attention to the material but were not allowed to take notes. Immediately following the video, participants had 20 min to complete the immediate post-test; all finished within this time frame. On the third day (occurring on the next school day after viewing the video and completing the immediate post-test), the participants were randomly assigned to either the visual or verbal explanation condition. The typed instructions were given to participants along with a blank 8.5 × 11 in. sheet of paper for their explanations. The instructions differed for each condition. For the visual condition, the instructions were as follows: “You have just finished learning about chemical bonding. On the next piece of paper, draw an explanation of how atoms bond and how ionic and covalent bonds differ. Draw your explanation so that another student your age who has never studied this topic will be able to understand it. Be as clear and complete as possible, and remember to use pictures/diagrams only. After you complete your explanation, you will be asked to answer a series of questions about bonding.”

For the verbal condition the instructions were: “You have just finished learning about chemical bonding. On the next piece of paper, write an explanation of how atoms bond and how ionic and covalent bonds differ. Write your explanation so that another student your age who has never studied this topic will be able to understand it. Be as clear and complete as possible. After you complete your explanation, you will be asked to answer a series of questions about bonding.”

Participants were instructed to read the instructions carefully before beginning the task. The participants completed their explanations as a whole-class activity. Participants were given unlimited time to complete their explanations. Upon completion of their explanations, participants were asked to complete the ten-question delayed post-test (comparable to but different from the first) and were given a maximum of 20 min to do so. All participants completed their explanations as well as the post-test during the 45-min class period.

## Results

### Spatial ability

The mean score on the MRT was 10.39, with a median of 11. Boys (M = 12.5, SD = 4.8) scored significantly higher than girls (M = 8.0, SD = 4.0), F(1, 125) = 24.49, *p* < 0.01. Participants were split into low and high spatial ability based on the median.

### Learning outcomes

**Fig. 5:**
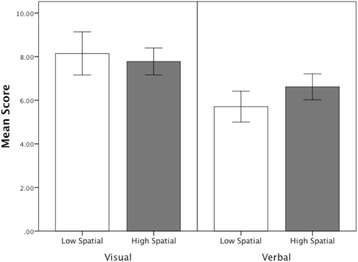
Scores on the post-tests by explanation type and spatial ability

The maximum score for both the immediate and delayed post-test was 10 points. A repeated measures ANOVA showed that the difference between the immediate post-test scores (M = 4.63, SD = 0.469) and delayed post-test scores (M = 7.04, SD = 0.299) was statistically significant F(1, 125) = 18.501, *p* < 0.05). Without any further instruction, scores increased following the generation of a visual or verbal explanation. Both groups improved significantly; those who created visual explanations (M = 8.22, SD = 0.208), F(1, 125) = 51.24, *p* < 0.01, Cohen’s *d* = 1.27 as well as those who created verbal explanations (M = 6.31, SD = 0.273), F(1,125) = 15.796, *p* < 0.05, Cohen’s *d* = 0.71. As seen in Fig. 5, participants who generated visual explanations (M = 0.822, SD = 0.208) scored considerably higher on the delayed post-test than participants who generated verbal explanations (M = 0.631, SD = 0.273), F(1, 125) = 19.707, *p* < 0.01, Cohen’s *d* = 0.88. In addition, high spatial participants (M = 0.824, SD = 0.273) scored significantly higher than low spatial participants (M = 0.636, SD = 0.207), F(1, 125) = 19.94, *p* < 0.01, Cohen’s *d* = 0.87. The results of the test of the interaction between group and spatial ability was not significant.

### Coding explanations

Explanations were coded for structural and functional content, arrows, specific examples, and multiple representations. A subset of the explanations (20%) was coded by both the first author and a middle school science teacher with expertise in Chemistry. Both scorers used the same coding system as a guide. The percentage of agreement between scores was above 90 for all measures. The first author then scored the remainder of the explanations. As evident from Fig. 4, the visual explanations were individual inventions; they neither resembled each other nor those used in teaching. Most contained language, especially labels and symbolic language such as NaCl.

#### Structure, function, and modality

Visual and verbal explanations were coded for depicting or describing structural and functional components. The structural components included the following: the correct number of valence electrons, the correct charges of atoms, the bonds between non-metals for covalent molecules and between a metal and non-metal for ionic molecules, the crystalline structure of ionic molecules, and that covalent bonds were individual molecules. The functional components included the following: transfer of electrons in ionic bonds, sharing of electrons in covalent bonds, attraction between ions of opposite charge, bonding resulting in atoms with neutral charge and stable electron shell configurations, and outcome of bonding shows molecules with overall neutral charge. The presence of each component was awarded 1 point; the maximum possible points was 5 for structural and 5 for functional information. The modality, visual or verbal, of each component was also coded; if the information was given in both formats, both were coded.

**Fig. 6:**
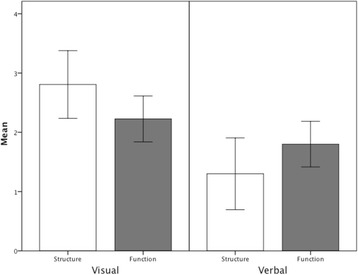
Average number of structural and functional components in visual and verbal explanations

As displayed in Fig. 6, visual explanations contained a significantly greater number of structural components (M = 2.81, SD = 1.56) than verbal explanations (M = 1.30, SD = 1.54), F(1, 125) = 13.69, *p* < 0.05. There were no differences between verbal and visual explanations in the number of functional components. Structural information was more likely to be depicted (M = 3.38, SD = 1.49) than described (M = 0.429, SD = 1.03), F(1, 62) = 21.49, *p* < 0.05, but functional information was equally likely to be depicted (M = 1.86, SD = 1.10) or described (M = 1.71, SD = 1.87).

**Fig. 7:**
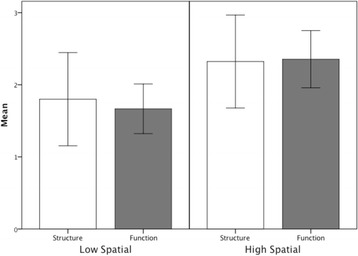
Average number of structural and functional components created by low and high spatial ability learners

Functional information expressed verbally in the visual explanations significantly predicted scores on the post-test, F(1, 62) = 21.603, *p* < 0.01, while functional information in verbal explanations did not. The inclusion of structural information did not significantly predict test scores. As seen Fig. 7, explanations created by high spatial participants contained significantly more functional components, F(1, 125) = 7.13, *p* < 0.05, but there were no ability differences in the amount of structural information created by high spatial participants in either visual or verbal explanations.

#### Arrows

Ninety-two percent of visual explanations contained arrows. Arrows were used to indicate motion as well as to label. The use of arrows was positively correlated with scores on the post-test, r = 0.293, *p* < 0.05. There were no significant differences in the use of arrows between low and high spatial participants.

#### Specific examples

Explanations were coded for the use of specific examples, such as NaCl, to illustrate ionic bonding and CO_2_ and O_2_ to illustrate covalent bonding. High spatial participants (M = 1.6, SD = 0.69) used specific examples in their verbal and visual explanations more often than low spatial participants (M = 1.07, SD = 0.79), a marginally significant effect F(1, 125) = 3.65, *p* = 0.06. Visual and verbal explanations did not differ in the presence of specific examples. The inclusion of a specific example was positively correlated with delayed test scores, r = 0.555, *p* < 0.05.

#### Use of multiple representations

Many of the explanations (65%) contained multiple representations of bonding. For example, ionic bonding and its properties can be represented at the level of individual atoms or at the level of many atoms bonded together in a crystalline compound. The representations that were coded were as follows: symbolic (e.g. NaCl), atomic (showing structure of atom(s), and macroscopic (visible). Participants who created visual explanations generated significantly more (M =1.79, SD = 1.20) than those who created verbal explanations (M = 1.33, SD = 0.48), F (125) = 6.03, *p* < 0.05. However, the use of multiple representations did not significantly correlate with delayed post-test scores on the delayed post-test.

#### Metaphoric explanations

Although there were too few examples to be included in the statistical analyses, some participants in the visual group created explanations that used metaphors and/or analogies to illustrate the differences between the types of bonding. Figure 4 shows examples of metaphoric explanations. In one example, two stick figures are used to show “transfer” and “sharing” of an object between people. In another, two sharks are used to represent sodium and chlorine, and the transfer of fish instead of electrons.

## Discussion

In the second experiment, students were introduced to chemical bonding, a more abstract and complex set of phenomena than the bicycle pump used in the first experiment. Students were tested immediately after instruction. The following day, half the students created visual explanations and half created verbal explanations. Following creation of the explanations, students were tested again, with different questions. Performance was considerably higher as a consequence of creating either explanation despite the absence of new teaching. Generating an explanation in this way could be regarded as a test of learning. Seen this way, the results echo and amplify previous research showing the advantages of testing over study (e.g. Roediger et al., 2011; Roediger & Karpicke, 2006; Wheeler & Roediger, 1992). Specifically, creating an explanation requires selecting the crucial information, integrating it temporally and causally, and expressing it clearly, processes that seem to augment learning and understanding without additional teaching. Importantly, creating a visual explanation gave an extra boost to learning outcomes over and above the gains provided by creating a verbal explanation. This is most likely due to the directness of mapping complex systems to a visual-spatial format, a format that can also provide a natural check for completeness and coherence as well as a platform for inference. In the case of this more abstract and complex material, generating a visual explanation benefited both low spatial and high spatial participants even if it did not bring low spatial participants up to the level of high spatial participants as for the bicycle pump.

Participants high in spatial ability not only scored better, they also generated better explanations, including more of the information that predicted learning. Their explanations contained more functional information and more specific examples. Their visual explanations also contained more functional information.

As in Experiment 1, qualities of the explanations predicted learning outcomes. Including more arrows, typically used to indicate function, predicted delayed test scores as did articulating more functional information in words in visual explanations. Including more specific examples in both types of explanation also improved learning outcomes. These are all indications of deeper understanding of the processes, primarily expressed in the visual explanations. As before, these findings provide ways that educators can guide students to craft better visual explanations and augment learning.

### General discussion

Two experiments examined how learner-generated explanations, particularly visual explanations, can be used to increase understanding in scientific domains, notably those that contain “invisible” components. It was proposed that visual explanations would be more effective than verbal explanations because they encourage completeness and coherence, are more explicit, and are typically multimodal. These two experiments differ meaningfully from previous studies in that the information selected for drawing was not taken from a written text, but from a physical object (bicycle pump) and a class lesson with multiple representations (chemical bonding).

The results show that creating an explanation of a STEM phenomenon benefits learning, even when the explanations are created after learning and in the absence of new instruction. These gains in performance in the absence of teaching bear similarities to recent research showing gains in learning from testing in the absence of new instruction (e.g. Roediger et al., 2011; Roediger & Karpicke, 2006; Wheeler & Roediger, 1992). Many researchers have argued that the retrieval of information required during testing strengthens or enhances the retrieval process itself. Formulating explanations may be an especially effective form of testing for post-instruction learning. Creating an explanation of a complex system requires the retrieval of critical information and then the integration of that information into a coherent and plausible account. Other factors, such as the timing of the creation of the explanations, and whether feedback is provided to students, should help clarify the benefits of generating explanations and how they may be seen as a form of testing. There may even be additional benefits to learners, including increasing their engagement and motivation in school, and increasing their communication and reasoning skills (Ainsworth, Prain, & Tytler, 2011). Formulating a visual explanation draws upon students’ creativity and imagination as they actively create their own product.

As in previous research, students with high spatial ability both produced better explanations and performed better on tests of learning (e.g. Uttal et al., 2013). The visual explanations of high spatial students contained more information and more of the information that predicts learning outcomes. For the workings of a bicycle pump, creating a visual as opposed to verbal explanation had little impact on students of high spatial ability but brought students of lower spatial ability up to the level of students with high spatial abilities. For the more difficult set of concepts, chemical bonding, creating a visual explanation led to much larger gains than creating a verbal one for students both high and low in spatial ability. It is likely a mistake to assume that how and high spatial learners will remain that way; there is evidence that spatial ability develops with experience (Baenninger & Newcombe, 1989). It is possible that low spatial learners need more support in constructing explanations that require imagining the movement and manipulation of objects in space. Students learned the function of the bike pump by examining an actual pump and learned bonding through a video presentation. Future work to investigate methods of presenting material to students may also help to clarify the utility of generating explanations.

## Conclusion

Creating visual explanations had greater benefits than those accruing from creating verbal ones. Surely some of the effectiveness of visual explanations is because they represent and communicate more directly than language. Elements of a complex system can be depicted and arrayed spatially to reflect actual or metaphoric spatial configurations of the system parts. They also allow, indeed, encourage, the use of well-honed spatial inferences to substitute for and support abstract inferences (e.g. Larkin & Simon, 1987; Tversky, 2011). As noted, visual explanations provide checks for completeness and coherence, that is, verification that all the necessary elements of the system are represented and that they work together properly to produce the outcomes of the processes. Visual explanations also provide a concrete reference for making and checking inferences about the behavior, causality, and function of the system. Thus, creating a visual explanation facilitates the selection and integration of information underlying learning even more than creating a verbal explanation.

Creating visual explanations appears to be an underused method of supporting and evaluating students’ understanding of dynamic processes. Two obstacles to using visual explanations in classrooms seem to be developing guidelines for creating visual explanations and developing objective scoring systems for evaluating them. The present findings give insights into both. Creating a complete and coherent visual explanation entails selecting the essential components and linking them by behavior, process, or causality. This structure and organization is familiar from recipes or construction sets: first the ingredients or parts, then the sequence of actions. It is also the ingredients of theater or stories: the players and their actions. In fact, the creation of visual explanations can be practiced on these more familiar cases and then applied to new ones in other domains. Deconstructing and reconstructing knowledge and information in these ways has more generality than visual explanations: these techniques of analysis serve thought and provide skills and tools that underlie creative thought. Next, we have shown that objective scoring systems can be devised, beginning with separating the information into structure and function, then further decomposing the structure into the central parts or actors and the function into the qualities of the sequence of actions and their consequences. Assessing students’ prior knowledge and misconceptions can also easily be accomplished by having students create explanations at different times in a unit of study. Teachers can see how their students’ ideas change and if students can apply their understanding by analyzing visual explanations as a culminating activity.

Creating visual explanations of a range of phenomena should be an effective way to augment students’ spatial thinking skills, thereby increasing the effectiveness of these explanations as spatial ability increases. The proverbial reading, writing, and arithmetic are routinely regarded as the basic curriculum of school learning and teaching. Spatial skills are not typically taught in schools, but should be: these skills can be learned and are essential to functioning in the contemporary and future world (see Uttal et al., 2013). In our lives, both daily and professional, we need to understand the maps, charts, diagrams, and graphs that appear in the media and public places, with our apps and appliances, in forms we complete, in equipment we operate. In particular, spatial thinking underlies the skills needed for professional and amateur understanding in STEM fields and knowledge and understanding STEM concepts is increasingly required in what have not been regarded as STEM fields, notably the largest employers, business, and service.

This research has shown that creating visual explanations has clear benefits to students, both specific and potentially general. There are also benefits to teachers, specifically, revealing misunderstandings and gaps in knowledge. Visualizations could be used by teachers as a formative assessment tool to guide further instructional activities and scoring rubrics could allow for the identification of specific misconceptions. The bottom line is clear. Creating a visual explanation is an excellent way to learn and master complex systems.

## Additional file

Additional file 1: Post-tests. (DOC 44 kb)
