# Supplementary material for: Creating visual explanations improves learning
Source: Cogn Res Princ Implic. 2016 Dec 7;1:27. doi: 10.1186/s41235-016-0031-6 (PMC5256450; doi:10.1186/s41235-016-0031-6)
Supplement: Supplementary file 1 — Post-tests. (DOC 44 kb) [file 41235_2016_31_MOESM1_ESM.doc]

Additional file 1: POST-TESTS

Experiment 1: Bicycle Tire Pump

**Bicycle Pump**

**True/False Statements**

**Instructions: On the line beside each statement, write a “T” if the statement is true or an “F” if the statement is false.**

_______ 1. The piston never touches the wall of the cylinder.

_______ 2. Pressure build-up in the cylinder chamber opens the outlet valve.

_______ 3. The piston is attached to the handle.

_______ 4. When the handle is pulled up, the inlet valve opens.

_______ 5. The outlet valve is at the top of the piston.

_______ 6. Air enters the chamber when the handle is pulled up.

_______ 7. The inlet valve closes when the handle is pushed down.

_______ 8. The inlet valve is open when the outlet valve is closed.

_______ 9. The downward movement of the piston causes the inlet valve to close.

_______ 10. The outlet valve is open when the piston is raised.

_______ 11. The outlet valve allows air to enter the chamber.

_______ 12. The pump will not work if the outlet valve stays open.

_______ 13. Next to the handle is the inlet valve.

_______ 14. Next to the hose is the outlet valve.

_______ 15. The outlet valve is in the middle of the chamber.

_______ 16. The inlet valve is inside the piston.

Experiment 2: Chemical Bonding Immediate Post-Test

**Instructions: On the line beside each statement, write the letter of the correct answer. For short answer questions, provide a complete and detailed answer.**

_______ 1. The electrons in a nonpolar covalent bond are

1. shared equally
2. shared unequally
3. gained
4. transferred

_______ 2. When Magnesium bonds, how many electrons are gained/lost and what is the charge on the ion that it forms?

1. loses 2 electrons to form a Mg ion with a 2- charge
2. gains 2 electrons to form a Mg ion with a 2- charge
3. loses 2 electrons to form a Mg ion with a 2+ charge
4. gains 2 electrons to form a Mg ion with a 2+ charge

_______ 3. What is the correct formula for the ion that has 11 protons and 10 electrons?

1. He
2. He+
3. Na+
4. Na

_______ 4. How many valence electrons does Al have?

1. 1
2. 2
3. 3
4. 0

_______ 5. How many valence electrons does Li have?

1. 1
2. 2
3. 3
4. 0

_______6. What type of bonds are formed in N_2_O_4_?

1. ionic
2. polar covalent
3. non-polar covalent
4. metallic

_______ 7. An atom becomes a positive ion when it

1. is attracted to all nearby atoms
2. gains an electron from another atom
3. loses an electron to another atom
4. shares an electron with another atom

8. Is Silicon Dioxide an example of ionic or covalent bonding? Explain.

9. Draw a diagram showing the bond between Calcium and Chlorine.

10. Draw a diagram showing the bond in Br_2_

Chemical Bonding Delayed Post-Test

**Instructions: On the line beside each statement, write the letter of the correct answer. For short answer questions, provide a complete and detailed answer.**

_______1. What happens in a polar covalent bond?

- 1. electrons are exchanged
  2. electrons are shared equally
  3. electrons are shared unequally
  4. protons are shared

_______2. In the compound NaCl, electrons are

- 1. shared equally
  2. shared but not equally
  3. transferred between atoms to form ions
  4. freely moving among the atoms

_______3. Compounds can be separated by

- 1. breaking the atoms into smaller pieces
  2. breaking the bonds between the atoms
  3. using a magnet to attract certain atoms
  4. evaporating the liquid that contains the atoms

_______4. What part(s) of atoms are involved in bonding?

- 1. the whole atom
  2. the nucleus
  3. electrons
  4. protons

_______5. How many electrons are in the outermost energy level of lithium?

- 1. 1
  2. 2
  3. 7
  4. 8

_______6. How many valence electrons does fluorine have?

- 1. 1
  2. 2
  3. 7
  4. 8

_______7. What types of bonds are in CO_2_?

- 1. ionic
  2. polar covalent
  3. non-polar covalent
  4. metallic

8. Draw a diagram showing the bond between Calcium and Fluorine.

9. Draw a diagram showing the bond in O_2_.

10. What would happen if lithium and fluorine and were combined chemically with each other?
